# Characterisation of a High-Performance Al–Zn–Mg–Cu Alloy Designed for Wire Arc Additive Manufacturing

**DOI:** 10.3390/ma13071610

**Published:** 2020-04-01

**Authors:** Paulo J. Morais, Bianca Gomes, Pedro Santos, Manuel Gomes, Rudolf Gradinger, Martin Schnall, Salar Bozorgi, Thomas Klein, Dominik Fleischhacker, Piotr Warczok, Ahmad Falahati, Ernst Kozeschnik

**Affiliations:** 1 Instituto de Soldadura e Qualidade, Av. Prof. Dr. Cavaco Silva, 33, 2740-120 Porto Salvo, Portugal; bfgomes@isq.pt (B.G.); pmsantos@isq.pt (P.S.); magomes@isq.pt (M.G.);; 2 LKR Light Metals Technologies Ranshofen, Austrian Institute of Technology, Lamprechtshausenerstraße 61, 5282 Ranshofen-Braunau, Austria; rudolf.gradinger@ait.ac.at (R.G.); martin.schnall@ait.ac.at (M.S.); salar.bozorgi@ait.ac.at (S.B.); thomas.klein@ait.ac.at (T.K.); 3 SinusPro GmbH, Conrad-von-Hötzendorf-Straße 127, 8010 Graz, Austria; dominik.fleischhacker@sinuspro.at; 4 MatCalc Engineering GmbH, Gumpendorfer Strasse 21, 1060 Vienna, Austria; piotr.warczok@mceng.at (P.W.); ahmad.falahati@tuwien.ac.at (A.F.); ernst.kozeschnik@tuwien.ac.at (E.K.); 5 Institute of Materials Science and Technology, TU Wien, Getreidemarkt 9/E308, 1060 Vienna, Austria

**Keywords:** wire arc additive manufacturing, precipitation hardening, Al–Zn–Mg–Cu alloys, microstructure characterisation, mechanical properties

## Abstract

Ever-increasing demands of industrial manufacturing regarding mechanical properties require the development of novel alloys designed towards the respective manufacturing process. Here, we consider wire arc additive manufacturing. To this end, Al alloys with additions of Zn, Mg and Cu have been designed considering the requirements of good mechanical properties and limited hot cracking susceptibility. The samples were produced using the cold metal transfer pulse advanced (CMT-PADV) technique, known for its ability to produce lower porosity parts with smaller grain size. After material simulations to determine the optimal heat treatment, the samples were solution heat treated, quenched and aged to enhance their mechanical performance. Chemical analysis, mechanical properties and microstructure evolution were evaluated using optical light microscopy, scanning electron microscopy, energy dispersive X-ray spectroscopy, X-ray fluorescence analysis and X-ray radiography, as well as tensile, fatigue and hardness tests. The objective of this research was to evaluate in detail the mechanical properties and microstructure of the newly designed high-performance Al–Zn-based alloy before and after ageing heat treatment. The only defects found in the parts built under optimised conditions were small dispersed porosities, without any visible cracks or lack of fusion. Furthermore, the mechanical properties are superior to those of commercial 7xxx alloys and remarkably independent of the testing direction (parallel or perpendicular to the deposit beads). The presented analyses are very promising regarding additive manufacturing of high-strength aluminium alloys.

## 1. Introduction

Additive manufacturing (AM) is a manufacturing technology which has been evolving at an enormous rate in the last 30 years. AM uses 3D CAD models to build parts by adding material successively, layer by layer, allowing the production of complex geometric shapes and topology optimised parts. The additive manufacturing of metals, which has become an area of extensive research work, is transforming many industrial sectors by reducing component lead time and material waste. Many different industries using metallic materials, such as aerospace, automotive, tooling, and health care, among others, are already taking advantage of AM [1,2]. Nevertheless, metallic parts produced by AM are susceptible to various defects, property degradation and compositional changes that need to be addressed [3].

Due to the high strength-to-weight ratio and ductility at room temperature, Al–Zn–Mg–Cu alloys are used in the automotive, aerospace, aircraft and competitive sport industries [4,5,6,7]. In these industries, new materials with enhanced mechanical properties are always needed to satisfy the ever-increasing demands for the weight reduction of structures and components. The strength of Al–Zn–Mg–Cu alloys is mainly controlled by the ageing process through the precipitation and growth of very fine precipitates of the η′-phase (a semi-coherent, metastable precursor of the equilibrium MgZn_2_ phase) evolving from the Zn and Mg Guinier–Preston (GP) zones [8,9,10]. The purpose of Cu in these alloys is to increase the ageing rate by increasing the degree of super-saturation, possibly, through nucleation of Al_2_CuMg (S-Phase) [11,12,13]. 

The ageing of Al–Zn–Mg–Cu alloys from room temperature to relatively low ageing temperatures is accompanied by the generation of GP zones with an approximately spherical shape [14]. With increasing ageing time, the GP zones increase in size and the strength of the alloy increases. Extended ageing at temperatures above room temperature transforms the GP zones in alloys with relatively high Zn and Mg ratios into the transition precipitates known as η′, T′ or S′, the precursor of the equilibrium MgZn_2_, T (Al_2_Mg_3_Zn_3_) and S (Al_2_CuMg)-phase precipitates [15,16,17].

The effect of alloying elements on welding additive processability and mechanical properties of Al–Zn–Mg–Cu alloys have been studied in different research works [18,19,20]. For example, the addition of Mn to Fe-containing Al–Zn–Mg–Cu alloys can promote the formation of the α-phase (Al_15_(Fe,Mn)_3_Si_2_) instead of the more harmful β-phases (Al_5_FeSi) [11,21,22,23]. Al–Zn–Mg alloys are known as materials difficult to cast and weld, due to subsequent hot cracking failures. This feature clearly creates a challenge to design alloys suitable for the wire arc additive manufacturing process. Analysis of the available hot tearing diagrams for this system indicated that alloys with a Mg:Zn ratio >1 are expected to have acceptable weldability [24]. Furthermore, alloys with this solute ratio might exhibit satisfactory mechanical properties [25,26]. The alloy compositions were designed based on intensive literature studies and theoretical thermodynamic considerations.

Among the manifold additive manufacturing techniques, technologies using metal powder can produce parts with high accuracy and comparably low deposition rates, which renders them more suitable to produce small parts with complex geometry [27]. When the powder is replaced by wire, higher deposition rates can be achieved, the costs with raw material are reduced, and the process is safer. Due to these benefits, the wire arc additive manufacturing process has been considered and many studies have been conducted using this technology [28,29,30,31,32]. Using this process, deposition rates up to 13-fold higher than in powder-based techniques can be achieved [33] and the investment costs in equipment are reduced when compared to, e.g., laser-based AM [34]. Wire arc additive manufacturing parts, however, present higher surface roughness, in the range of approximately 500 µm [27], which could be reduced by process control. Cold metal transfer (CMT) is a modified gas metal arc welding (GMAW) process developed by Fronius. Characteristic for the CMT technology is a low heat input and splash-free welding. Porosity can stem from wire or substrate contaminants or from volatile alloying elements. These contaminants are cracked by the high energy of the electric arc, resulting in gas porosity that is trapped upon solidification and can hardly be reduced during processing [35]. The characteristic curve used (arc) is a mix of CMT pulses and alternating voltage (CMT-PADV), which has been shown to be well suited for wire arc additive manufacturing of aluminium [36]. 

The aim of the present work was to understand the influence of the wire arc process and new alloying system on the mechanical properties of the produced parts before and after the heat treatment, including detailed microstructural characterisation.

## 2. Materials and Methods 

### 2.1. Alloy Composition and Material Processing

The experimental alloy composition was cast using vertical continuous casting. The resultant chemical composition is given in Table 1. Subsequently, cylindrical preforms with a diameter of 35 mm and a length of 100 mm were machined and heated in a furnace to 435 °C. These preforms were then extruded to wires with a diameter of 1.6 mm and a length of approximately 4 m. The resultant segments were then joined and, after visual inspection, coiled, thus enabling wire arc experiments with novel wires with specified non-commercial chemical compositions and a small lot size.

**Figure 1:**
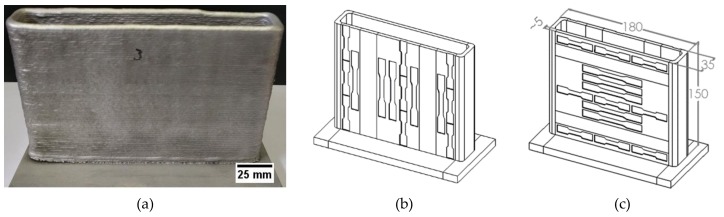
(**a**) Rectangular-shaped specimen produced by wire arc additive manufacturing on a flat substrate; (**b**) transverse tensile specimens; (**c**) longitudinal tensile specimens. All dimensions in mm.

The additive manufactured samples were produced with a CMT Advanced 4000 R (Fronius International GmbH, Wels, Austria) and a robot arm (ABB, Zurich, Switzerland) using CMT pulse advanced (PADV). Rectangular-shaped parts of approximately 170 × 30 × 120 mm^3^ (l × w × h) and an R equal to 5 mm for the corners were manufactured (Figure 1). A water-cooled base plate was used. The cleaning of the substrate before the deposition of the first layer and between layers was conducted with a stainless steel brush. The shielding gas used was argon. 

For further analyses, three different material conditions were investigated in detail: (i) the as-built (AB) material condition; (ii) the T6 material condition, which was obtained by a solution heat treatment at 470 °C for 5 h, followed by rapid cooling in water and a subsequent ageing treatment at 120 °C for 24 h; and (iii) the T73 material condition, which was obtained by a solution heat treatment at 470 °C for 5 h, followed by rapid cooling in water and a subsequent two-stage ageing treatment at 120 °C for 24 h, and then 160 °C for 24 h.

### 2.2. Characterisation Techniques

**Figure 2:**
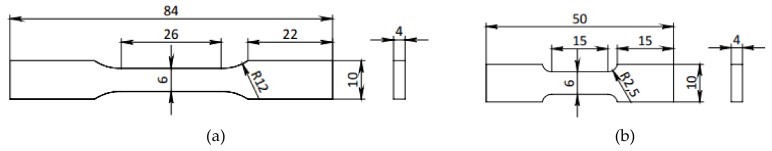
Tensile specimens: (**a**) regular size; (**b**) subsize. All dimensions in mm.

Tensile test specimens were extracted from the samples in the three different material conditions, in both transverse and longitudinal directions (Figure 1(b) and (c)). Standard specimens, according to ISO 6892-1:2016, were used to determine the average mechanical properties of the alloys in the different heat treatment conditions and direction (Figure 2a). Subsized specimens (Figure 2b) were used to probe local properties in the sample different regions (bottom, middle or top of the sample), and thus, to evaluate the homogeneity of the mechanical properties along the height and width of the produced parts. Three to five standard size specimens were tested for each material, heat treatment and conditions, whereas six to nine subsized specimens scattered over the whole face of the sample were tested for local properties determination (Figure 1b,c). 

**Figure 3:**
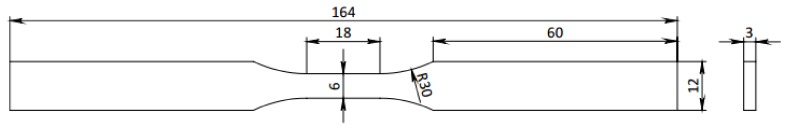
Fatigue specimen. All dimensions in mm.

Fatigue test specimens were extracted from the T73 tempered material condition in the longitudinal and transverse direction. The test conditions and specimen geometry (Figure 3) were in accordance with ASTM E466 standard. The specimen gauge length was further polished down to grade 1000 emery paper prior to fatigue testing. The tests were performed on a servo hydraulic machine under load control (Instron 8502, Norwood, Massachusetts, USA). Based on the tensile test results obtained for this material, a yield stress of 330 MPa was assumed and the maximum stress for fatigue tests set up in the range of 95% to 45% of the yield. All tests were performed with a stress ratio R = 0.1, commonly used in aeronautical and structural testing, a sinusoidal wave form and a frequency of 20 Hz. All tests were run up to failure—after which, the fracture surface was observed using a scanning electron microscope (JEOL JSM–6500F, Tokyo, Japan) to identify any features associated with the fracture initiation and propagation processes.

The transverse parts in the various conditions mentioned above were cold mounted, grinded with SiC paper and polished with diamond paste. Microhardness measurements were performed with a hardness tester (Mitutoyo Akashi AVK-C0, Kawasaki, Japan), using the Vickers method, with a load of 9.81 N and indentation time of 15 s. These measurements were performed in the centreline of the specimens along its height (one specimen along the height of the produced walls per condition was tested). Microstructure analysis was performed by optical light microscopy (Carl Zeiss Axiotech 100HD-3D, Oberkochen, Germany) and stereoscopy (Olympus SZX7, Tokyo, Japan). Specimens were electrochemically etched with Barkers Reagent (5 mL HBF4 (48%) + 200 mL water) using 15 V for 90–240 s. Scanning electron microscopy (SEM) and energy dispersive X-ray spectroscopy (EDS) results were obtained from the samples in as polished condition using a JSM–6500F microscope (JOEL, Tokyo, Japan) fitted with an EDS detector (Oxford Instruments X-Max^N^, Abingdon, UK).

Porosity analysis was performed in the as-polished samples, considering the three different regions and conditions presented above. Five images were obtained randomly for each region and evaluated using ImageJ software (National Institutes of Health, Bethesda, Maryland, USA) [37]. First, the scale was set in the software and, then, the image was thresholded carefully to ensure all the porosities remained in the image. Thus, all the particles with a minimum of 0.5 circularity were detected and measured by the software. A second analysis was performed to evaluate whether “non-pores” were measured and to measure the pores not detected. The porosity diameter considered was the average of width and height values of the smallest rectangle enclosing the selected pore. In addition, porosity was also analysed by X-ray radiography with X-ray equipment (YXLON ANDREX RIX-02, Hudson, Ohio, USA). The X-ray inspection followed the procedure prescribed by ISO 10675-2 standard.

In the grain measurement, the standard ASTM E112–2013 was used as a reference to establish the procedure. The number of grains in the specimens and respective areas were calculated using ImageJ software based on the visual counting performed in the etched micrographs. The images evaluated were of 200x or 500x magnification depending on the grain size. The ratio of the grains per area were then calculated based on the numbers of grain counted in a known area. To compare all the results, the average number of grains per 10⁶ μm^2^ was considered. In most cases, five images were evaluated for each condition.

### 2.3. Simulation of the Ageing Response

MatCalc software (MatCalc Engineering, Vienna, Austria) version 6.02 with the corresponding thermodynamic, mobility and physical databases for Al alloys was used for material simulations. The precipitation sequence during ageing was modelled in a simplified manner by considering Cu–Mg clusters and AlMgZn GP zones, nucleating directly from the FCC Al precipitation domain (bulk nucleation sites), while T-phase and S-phase precipitates were formed by a transformation of clusters and GP zones. Ageing treatments with various heat treatments were simulated, taking the annealed material at 500 °C as the initial material condition for precipitation kinetics simulations. For the validation of the results observed, experimentally, samples from the wire arc deposition process were solution heat treated to a peak temperature of 500 °C for 20 min. After quenching, three samples were subjected to an ageing process: (i) at 90 °C for 24 h, (ii) at 140 °C for 24 h and (iii) pre-aged at 90 °C for 24 h and aged at 140 °C for 24 h afterwards. Brinell hardness measurements were conducted in various time intervals during ageing. The chemical composition considered for the simulation was based on the chemical analysis of the alloy obtained after deposition. 

## 3. Results and Discussion

### 3.1. Wire Arc Deposition

In many instances, the limiting factor with regard to manufacturing is the capability of the alloys to be shaped into components. Consequently, there is a continuous demand to improve existing alloys, as well as to design new ones, in order to meet the requirements of various processing technologies. In the design of the alloy investigated in the present work, engineering properties such as weldability, hot-tearing susceptibility as well as resulting mechanical properties were in focus as detailed by Schnall et al. [38]. The chemical composition of the Al–Zn–Mg–Cu alloy was, thereby, iteratively adapted using a set of thermodynamic calculations, experimental determination of thermophysical properties and engineering tests of the hot-tearing susceptibility.

The absence of visible cracking, major porosity and no macroscopic shrinkage is a good indication of the process stability and the apparent good weldability of the newly designed alloy. Visual inspection of the specimens’ surfaces (Figure 1) suggests a low level of surface waviness, which is beneficent if the final surface needs to be machined, i.e., a lower buy-to-fly ratio is achieved or it lowers possible notch effects at unmachined surfaces of the final component. It is noted that the geometric features of the wire arc additive manufacturing specimens were geometrically consistent during consecutive manufacturing of several samples, suggesting uniform deposition conditions with a high reproducibility.

The arc’s colour during deposition appeared cyan, qualitatively suggesting the burn off of volatile chemical species. In Table 2, the chemical composition of the specimen after wire arc deposition also evidences the burn off of Mg and Zn during the additive manufacturing process. Hence, it is concluded that when aiming at a specified chemical composition of the final component, the wire needs to be over-alloyed with all volatile chemical elements.

**Figure 4:**
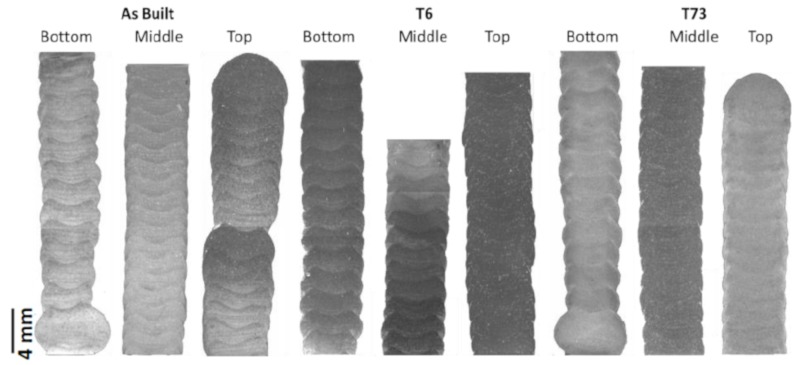
Macrographs of the transverse parts extracted from the samples in various material conditions.

Based on the macrographic results (Figure 4), it can be seen that the middle and top layers exhibited lower waviness, which may be explained by the higher temperature of the material in the deposition of these layers [39]. A higher temperature, together with the concomitant reduced viscosity, results in a higher flow-capability of the melt. Furthermore, the only discontinuities found in the micrographic images are pores. X-ray radiography indicated few dispersed porosities smaller than 1 mm in diameter. The general results presented so far demonstrate that modest adaptations in alloy composition enable improved weldability, allowing for crack-free fabrication of samples by wire arc additive manufacturing, potentially also enabling conventional welding of 7xxx alloy sheets, which will be the subject of further research.

**Figure 5:**
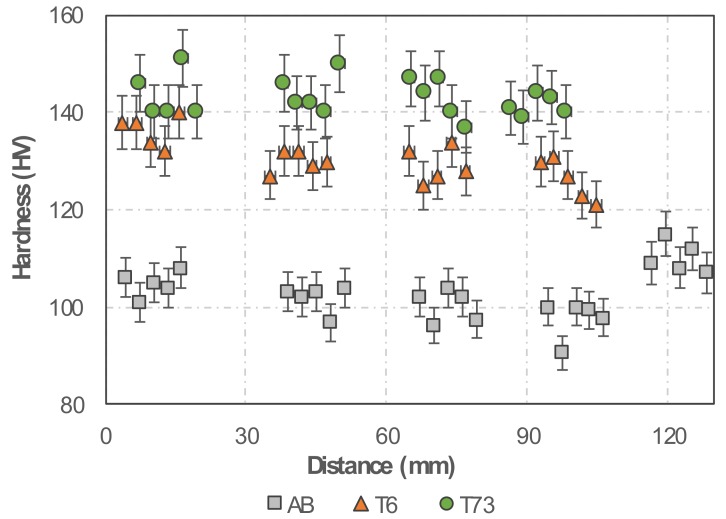
Evolution of Vickers hardness from the bottom (first layer) to the top of the wall, for three different material conditions (as-built, T6 and T73).

A constant level of hardness is seen along the height of the walls, where the average values found for samples in the AB, T6 and T73 conditions are 103, 131 and 143 HV, respectively (Figure 5). The constant hardness in each material condition suggests sufficient chemical homogeneity after wire arc processing, thus enabling a homogeneous hardening precipitate structure. It is noted that homogeneous mechanical properties counteract strain localisation upon loading, which is beneficial in case of highly loaded structural applications.

### 3.2. Microstructure Characterisation

#### 3.2.1. Microstructure Evolution

**Figure 6:**
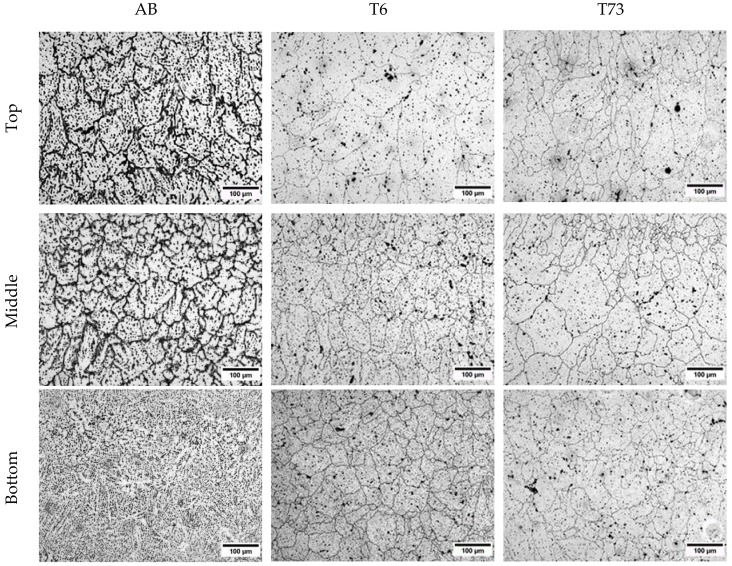
Micrograph of the samples in the AB, T6 and T73 conditions prepared using Barkers reagent.

When evaluating the micrographs of the deposited samples (Figure 6), it can be seen in the bottom region of the as-built condition that a dendritic structure is predominant, and no clear grain structure is revealed. In all the other conditions, a mixture of equiaxed and elongated grains are seen. It can be noted that a smaller equiaxed grain structure is discernible in the interlayer region, followed by elongated grains and a zone of coarser grains with mostly equiaxed grains. This is a common feature of aluminium processed by directed energy deposition due to solidification conditions within a pronounced thermal gradient [40].

#### 3.2.2. Porosity Evaluation

**Figure 7:**
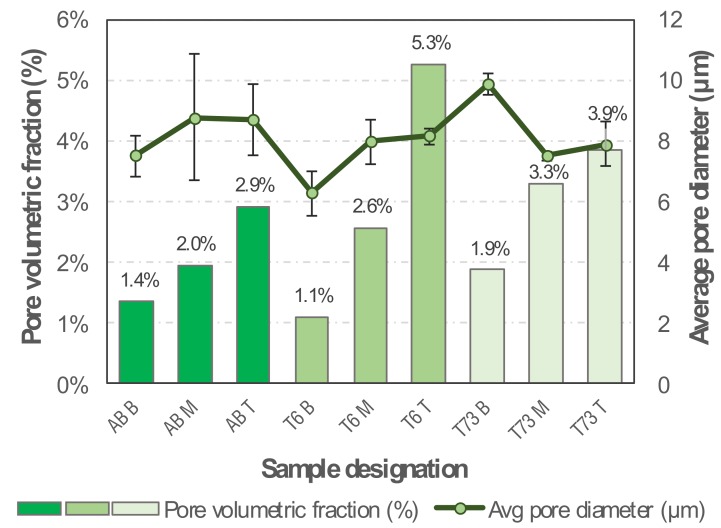
Pore volumetric fraction and average diameter of pores of the samples in the AB, T6 and T73 conditions.

The pore volumetric fraction analysis shows that this parameter increases along the samples’ height from the bottom to the top (Figure 7). This result might be related to the thermal gradient that is created during the wire arc deposition process. As the specimen’s height increases, so does the mean temperature, which affects the metallurgical processes occurring in the melt zone to a yet unknown extent. The smallest average pore diameter found was 6 µm and the highest was 10 µm. The variations among the three types of conditions (as-built, T6 and T73) are expected, since the heat treatments usually promote not only the increase in secondary porosity volume but also the coalescence of small micropores [41]. According to Gu et al. [42], some mechanisms responsible for micropores growth in Al alloys are Ostwald ripening [43], nucleation of new pores and growth of secondary pores [44]. When correlating these results to the results from the tensile test, performed with the subsize specimens extracted from the transverse direction, no visible relation is clearly identified (Figure 12), suggesting that the pores observed are sufficiently small and few as to not to interfere within the elastic deformation regime and do not cause premature failure upon plastic deformation (see Section 3.4). 

#### 3.2.3. Grain Number

**Figure 8:**
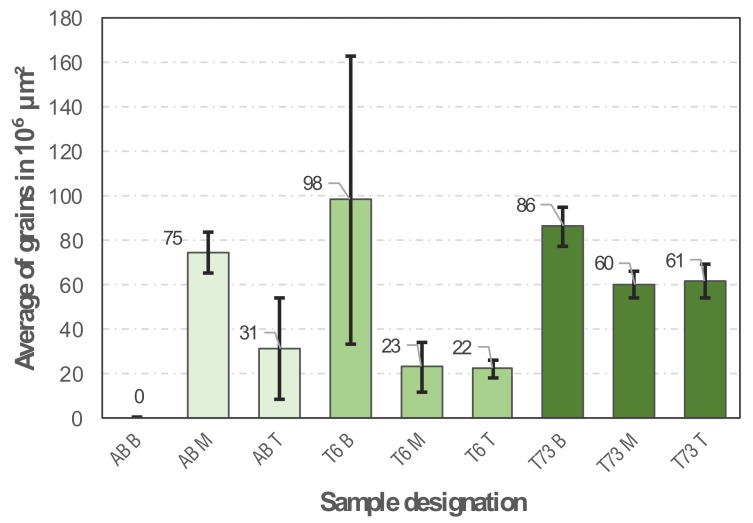
Average of grains in 10^6^ µm^2^ of the samples in the AB, T6 and T73 conditions.

When evaluating the number of grains in the different regions (bottom, middle and top) of the sample in the T6 and T73 conditions (Figure 8), it can be seen that the grains are smaller (higher in quantity) in the bottom region (it was not possible to measure any grain structure in the sample in the as-built condition in the bottom region due to this regions particular microstructure as visible in Figure 6). This observation underpins our previous argumentation: as the specimen height increases, the mean specimen temperature is increased. Thus, thermally controlled coarsening reactions prevail and dominate the resultant grain size via grain growth.

### 3.3. Heat Treatment Simulation

**Figure 9:**
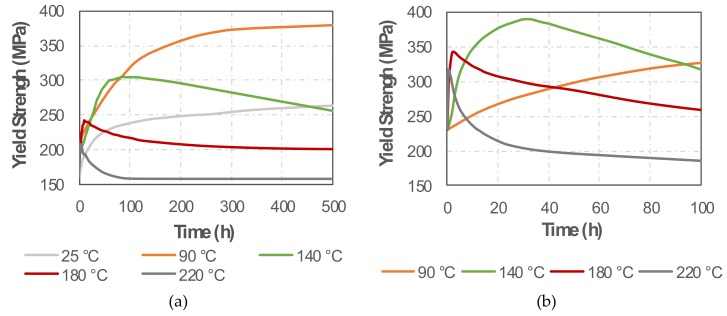
Simulated yield strength for various ageing treatments at various temperatures of: (**a**) single-stage treatment; (**b**) second treatment of double-stage treatment after ageing at 90 °C for 24 h.

In order to estimate the effects of the various heat treatments on the mechanical properties, MatCalc simulations were performed as described in subchapter 2. The maximum yield strength within the simulated time frame of 500 h was observed for ageing at 90 °C, when the material approaches a value of 380 MPa, as can be seen in Figure 9a. Application of lower temperatures resulted in lower yield strength values within the simulated time frame of 500 h. Application of higher temperatures resulted in earlier occurrence of the peak ageing response but in all cases below the strength value found for ageing at 90 °C. The application of a two-stage ageing treatment consisting of 24 h ageing at 90 °C followed by 32 h ageing at 140 °C resulted in the replication of the strength peak at 380 MPa, as shown in Figure 9b. It was found that the application of higher temperatures for the second ageing step resulted in faster coarsening of the initial precipitates, leading to lower strength peaks and faster over-ageing. Application of lower temperatures for the second ageing (i.e., 90 °C) did not increase the simulated strength peak, resulting only in its delayed occurrence.

**Figure 10:**
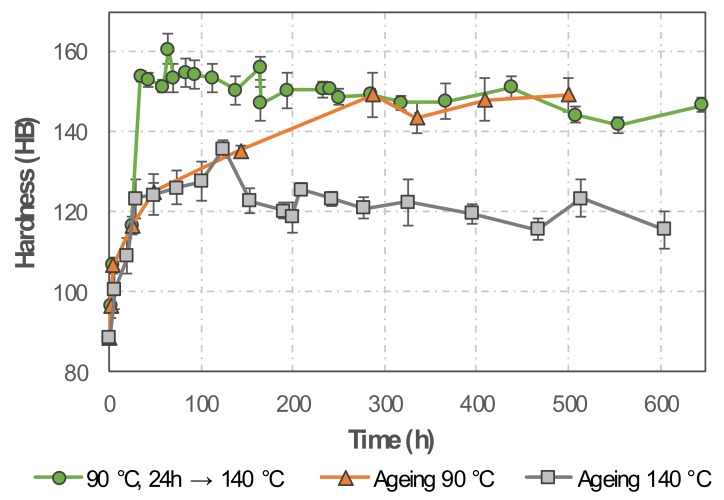
Brinell hardness evolution in samples subjected to various ageing treatments.

For validation of the simulation results, Brinell hardness tests were conducted and compared to the simulated yield strength for three different conditions (Figure 10). For the sample aged at 90 °C, the hardness increases slowly and reaches a plateau at 145–150 HB after ~275 h without any indication of over-ageing. A hardness peak at 130 HB was found for the sample aged at 140 °C after 125 h of ageing, showing a slow hardness decrease for longer ageing duration. The sample subjected to two-stage ageing treatment, held first at 90 °C for 24 h, followed by ageing at 140 °C, exhibited a notable hardness increase within 10 h of the second ageing treatment reaching the value ~155 HB. Within 100 h of the second stage ageing, the sample hardness remained above 150 HB, decreasing slowly afterwards. It is concluded that the experimentally observed trends satisfactorily underpin the simulation results and fit well to results of a recent work [9].

### 3.4. Mechanical Properties

**Figure 11:**
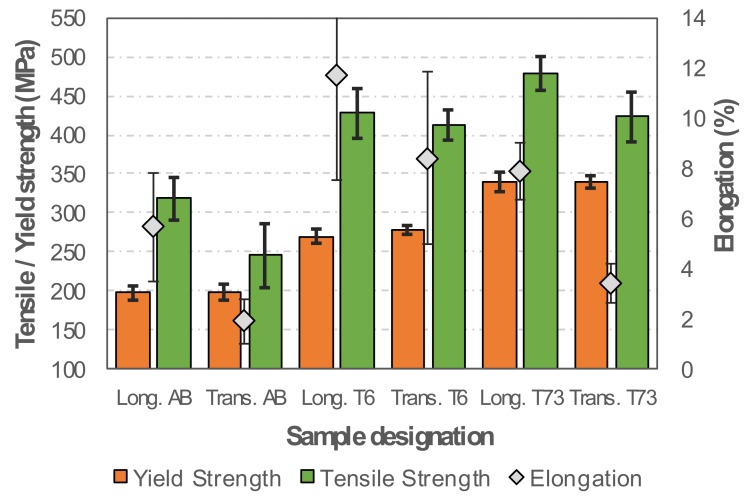
Average yield strength, tensile strength and elongation of samples in the AB, T6 and T73 conditions.

Concerning the deposition direction in the AB, T6 and T73 conditions, the tensile strength and elongation are lower in transverse specimens, indicating a certain degree of anisotropy (Figure 11). It should be noted that the yield strength is nearly isotropic, which is highly important for practical applications. It can be seen that the ageing treatment increases yield strength, tensile strength and elongation. The T73 condition presents the highest yield strength, while T6 presented a good combination of strength and ductility. When comparing the results obtained in this research with the designed alloy to various Al alloys used in the selective laser melting (SLM) the good mechanical properties achieved are noticeable [45].

**Figure 12:**
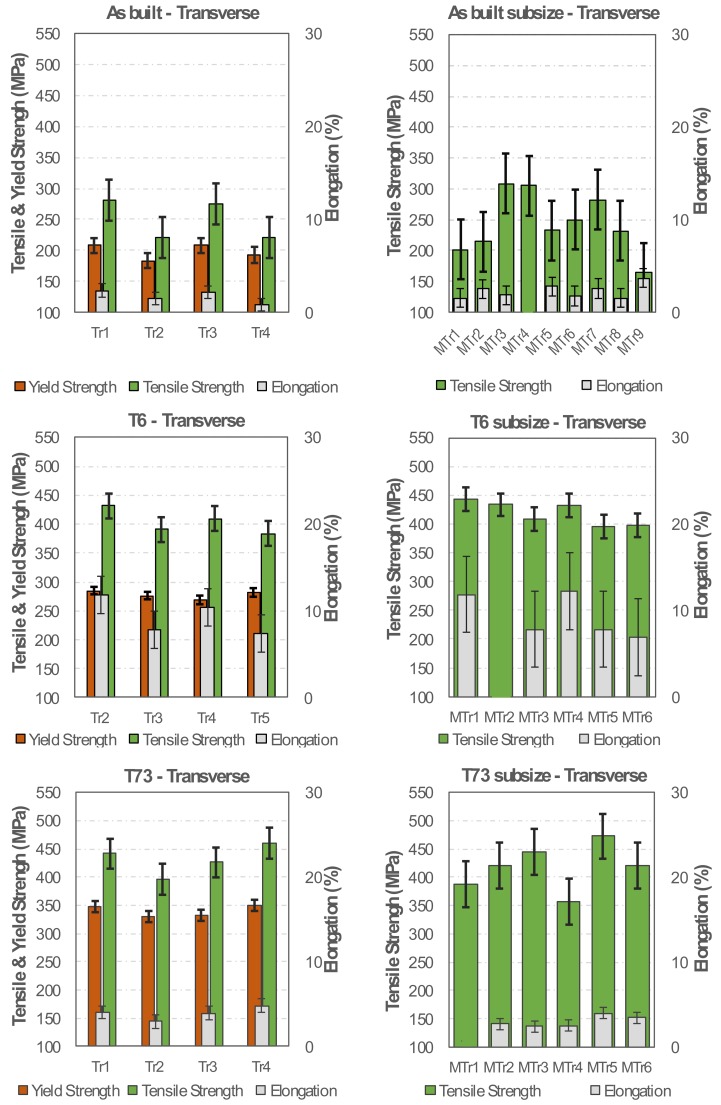
Yield strength, tensile strength and fracture elongation of the regular and subsize specimens in the AB, T6 and T73 conditions.

In the AB, T6 and T73 conditions, the tensile strength and the fracture elongation for standard and subsize specimens are similar, indicating homogenous mechanical properties along the height of the produced samples, as indicated by the mechanical characteristics of the analysed specimens taken in transverse direction (Figure 12).

**Figure 13:**
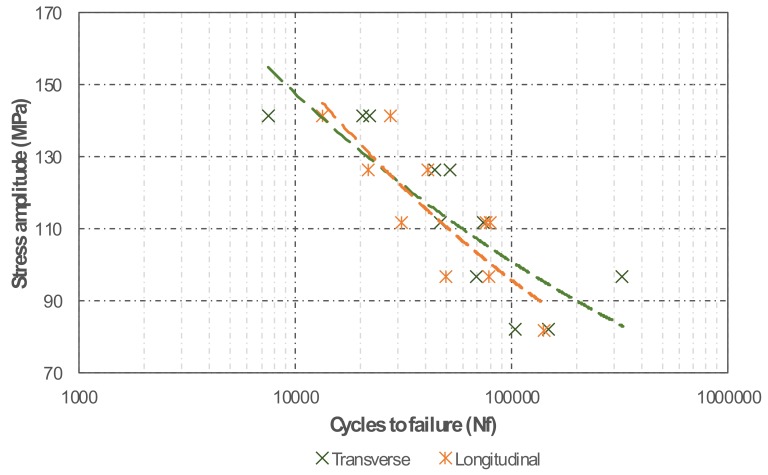
Wöhler curves of longitudinal and transverse specimens in the T73 condition parts.

Due to the limited amount of material available, fatigue testing was performed on specimens in the T73 condition only. This testing was intended to compare the material behaviour in the transverse and longitudinal directions, thus to obtain some indications on the performance of the manufacturing process, rather than fully characterise the fatigue behaviour of the material. The fatigue test results exhibited a considerable scatter for both deposition directions (Figure 13), particularly at higher stress levels. This scatter can presumably be explained by material structural heterogeneities and residual porosity. 

**Figure 14:**
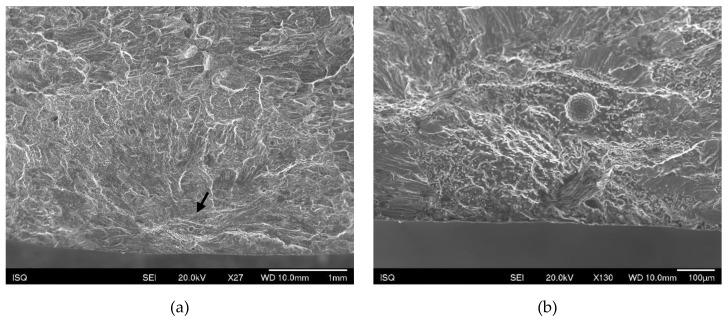
(**a**) A small pore in the initiation site of a longitudinal specimen; (**b**) detail of (a).

**Figure 15:**
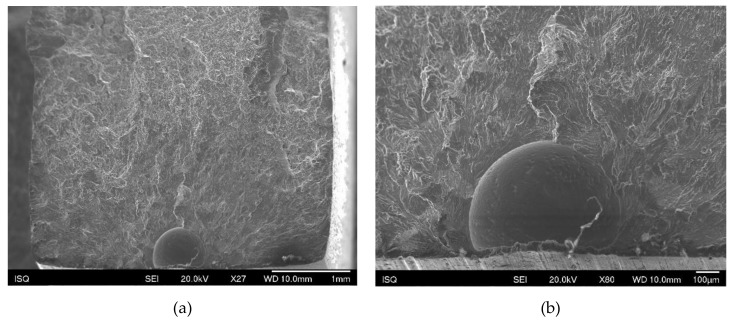
(**a**) A large subsurface pore in the initiation site of a perpendicular specimen; (**b**) detail of (a).

The main fracture initiation site feature evidenced in almost all specimens analysed was porosity (Figure 14). Post-test analysis of the fracture surfaces revealed that fracture often initiated from porosities at or close to the specimen surface. In some instances, these defects were previously internal or subsurface defects that emerged near the surface as a result of specimen machining and polishing (Figure 15).

**Figure 16:**
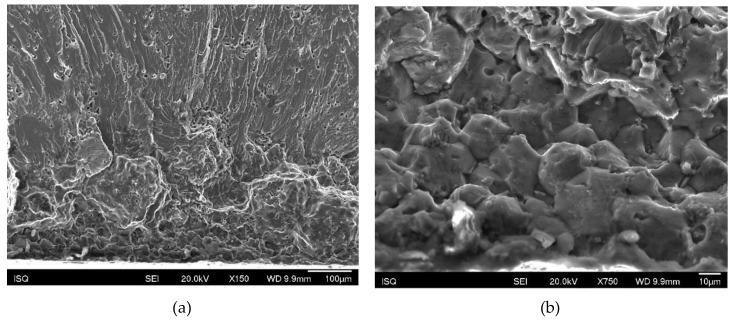
(**a**) Initiation site of a perpendicular specimen; (**b**) detail of the region between the pore and the surface showing intergranular decohesion.

Some intergranular brittleness was also observed, possibly as a result of minor oscillations of deposition conditions leading to some fatigue endurance reduction. This intergranular fracture mode seemed more frequent in perpendicular specimens than in longitudinal ones (Figure 16).

**Figure 17:**
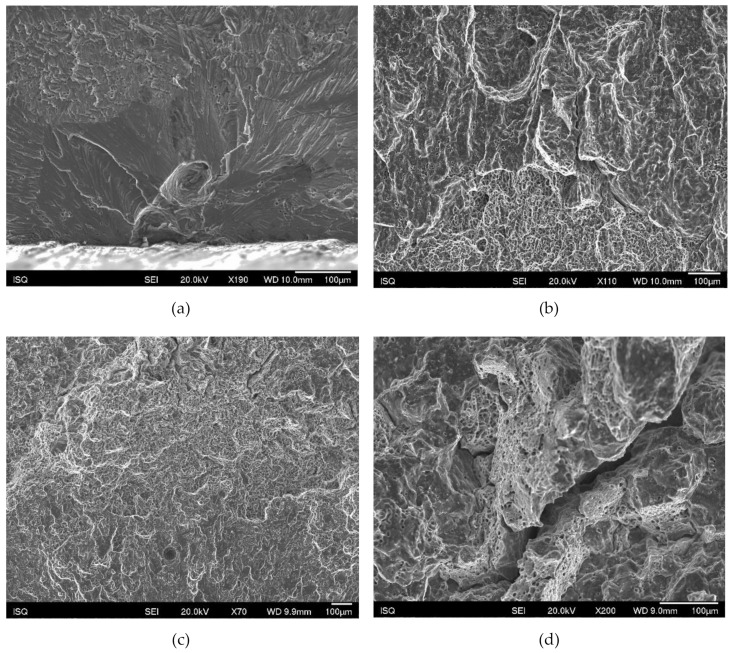
(**a**) Cleavage associated with porosity at the initiation site; (**b**) transition region; (**c**) propagation path with dimpled structure and pores; (**d**) propagation path with intergranular decohesion and dimpled structure.

Besides intergranular brittleness, the initiation region and earlier crack growth was often associated with cleavage fracture of grains shifting in a later propagation stage and final unstable fracture to dimpled ductile fracture. Again, porosity was often observed in the propagation path as well as intergranular decohesion, more frequent in the perpendicular specimens (Figure 17).

Despite the differences in the fracture behaviour, the material shows an almost identical behaviour in both longitudinal and transverse directions, which precludes potential for future use in mechanical parts. Comparison of fatigue data is difficult given the large variation of heat treatments, materials and testing conditions. The small number of fatigue tests performed in the present work also contribute to impair such comparisons. Nevertheless, the present results evidence the processability of 7xxx aluminium alloys by wire arc additive manufacturing with remarkable mechanical properties [46,47,48].

## 4. Conclusions

From the performed analyses of the novel Al–Zn–Mg–Cu alloy with improved processability, the following major conclusions can be drawn:

- Wire arc additive manufacturing of the Al5–Mg3–Zn–Cu alloy resulted in a component with exceptionally high mechanical strength when compared to other Al alloys. In fact, the achieved mechanical properties are even superior to the values available for many commercial 7xxx alloys. Furthermore, the heat treatment simulations indicate that even higher mechanical properties can be achieved using optimised ageing treatments.
- Concerning defects, the manufactured parts only exhibit few dispersed porosities and volumetric pore fraction changes along the height of the sample. The volumetric pore fraction is smaller in the bottom and bigger in the top region, while the grains are smaller in the bottom and bigger in the upper region. Both observations are in line with an increasing mean component temperature with an increasing height. Equiaxed and elongated grains are seen along the sample—a typical feature of wire arc processed aluminium. It can be noted that smaller equiaxed grains are seen in the interlayer region, followed by elongated grains and a zone of coarser grains with mostly equiaxed grains.
- The fatigue results showed a high scatter caused mainly by residual porosity, which was the main feature associated with failure initiation and eventual risk of intergranular brittleness.
- The high strength reached under 60 h of ageing time can be attributed to the two-stage ageing treatment, which promoted full formation of precipitation and growth of very fine precipitates of the η′ or T′-phase (semi-coherent, metastable precursors of the equilibrium MgZn_2_ or T (Al_2_Mg_3_Zn_3_) phases, respectively) from GP zones.
- The hardness measurements performed on the aged samples deposited by the wire arc process confirmed the simulation trends and the advantages of the two-stage ageing treatment as a processing method for this material.

This study demonstrates that the wire arc additive manufacturing of a novel 7xxx alloy is not only feasible but results in very good mechanical properties following ensuing heat treatments.

## Figures and Tables

**Table 1:** Chemical composition of the alloy in the as-cast condition measured by optical emission spectroscopy (OES) in weight percent.

As-cast (OES)	Al	Mg	Zn	Mn	Si	Cu	Fe	Cr	Ti	Zr
wt.%	89.30	5.87	3.58	0.49	0.07	0.33	0.11	0.04	0.05	0.12

**Table 2:** Chemical composition of the deposited alloy in the as-built condition measured by OES in weight percent.

As-built (OES)	Al	Mg	Zn	Mn	Si	Cu	Fe	Cr	Ti	Zr
wt.%	90.00	5.33	3.44	0.49	0.07	0.31	0.11	0.04	0.06	0.12
